# Experimental and Numerical Characterization of a Hybrid Fabry-Pérot Cavity for Temperature Sensing

**DOI:** 10.3390/s150408042

**Published:** 2015-04-07

**Authors:** Aitor Lopez-Aldaba, Ana Margarida Rodrigues Pinto, Manuel Lopez-Amo, Orlando Frazão, José Luís Santos, José Manuel Baptista, Hardy Baierl, Jean-Louis Auguste, Raphael Jamier, Philippe Roy

**Affiliations:** 1 Department of Electric and Electronic Engineering of the Public University of Navarra, 31006 Pamplona, Navarra, Spain; E-Mails: aitor.lopez@unavarra.es (A.L.-A.); mla@unavarra.es (M.L.-A.); 2 INESC P&D Brasil, 11055-300 Santos, São Paulo, Brazil; 3 INESC Porto, 4150-179 Porto, Portugal; E-Mails: ofrazao@fc.up.pt (O.F.); josantos@fc.up.pt (J.L.S.); jmb@inescporto.pt (J.M.B.); 4 Competence Center of Exact Sciences and Engineering of the University of Madeira, 9000-082 Funchal, Portugal; 5 University of Limoges, CNRS, Xlim, UMR7252, F-87000 Limoges, France; E-Mails: hardy.baierl@ipht-jena.de (H.B.); jean-louis.auguste@xlim.fr (J.-L.A.); raphael.jamier@xlim.fr (R.J.); philippe.roy@xlim.fr (P.R.)

**Keywords:** birefringence, fiber sensor, microstructured fiber, photonic crystal fiber, temperature sensor

## Abstract

A hybrid Fabry-Pérot cavity sensing head based on a four-bridge microstructured fiber is characterized for temperature sensing. The characterization of this cavity is performed numerically and experimentally in the L-band. The sensing head output signal presents a linear variation with temperature changes, showing a sensitivity of 12.5 pm/°C. Moreover, this Fabry-Pérot cavity exhibits good sensitivity to polarization changes and high stability over time.

## 1. Introduction

Optical fibers (OF) are efficient solutions for sensing due to their high sensitivity, small size, robustness, flexibility, ability for remote monitoring and multiplexing. Also of great importance for sensing applications is OF’s aptitude for operational work in the presence of unfavorable environmental conditions such as strong electromagnetic-fields, nuclear radiation, explosive or chemically corrosive media, as well as high temperatures. In these characteristics lies the recipe for the success of optical fiber sensing systems: in undertaking difficult measurement situations where the use of conventional electrical sensors is not adequate.

Optical fiber temperature sensors are very useful in different areas of application such as glass productions, furnaces of all sorts, high temperature processing, chemical industries, power generation, as well as in civil, aerospace and military industries. This type of OF sensor is one of the most required in the commercial market due to its vast number of applications [1].When microstructured optical fibers (MOFs) were first developed, it was quite clear that they were going to be able to improve the characteristics and potential of OF in the sensing field. MOFs geometry is characterized by a periodic arrangement of air-holes running along the entire length of the fiber centered on the core. The presence of air-holes in the cladding offers the opportunity to create dramatic morphologic changes, which leads to enhanced characteristics (transmission, birefringence, non-linearity, dispersion, *etc.*) as well as to the ability to manufacture a single material optical fiber [2,3]. Several MOF based temperature sensors have been made using different approaches. A 0.1636 nm/°C sensitive temperature sensor was obtained by the deposition of quantum dots nano-coatings, using the Layer-by-Layer technique, in the inner holes of an all-silica large mode area endlessly single mode MOF [4]. The inscription of long period gratings in a solid-core MOF showed a sensitivity of 10.9 pm/°C to temperature [5]. A polarimetric interrogation of a highly birefringent (Hi-Bi) MOF showed a sensitivity of 0.136 rad/°C at 1310 nm [6]. Even distribute temperature sensing was accomplished by using the birefringent effect of a transient Brillouin grating in a polarization-maintaining MOF, leading to a sensitivity of 23.5 MHz/°C [7]. Several interferometric configurations were used: an in-line fully liquid-filled MOF was used in a Mach-Zehnder interferometer with a sensitivity of 1.83 nm/°C [8]; by tapering a silica-core MOF, a modal interferometer was obtained with a sensitivity of 12 pm/°C for measurements up to 1000 °C [9]; and, using a two-hole birefringent MOF filled with metal indium in a Sagnac interferometric configuration it was possible to achieve a sensitivity of 6.3 nm/K [10].

Another approach for temperature sensing is to use Fabry-Pérot (FP) fiber interferometers. FP interferometers are a popular sensor configuration due to its sensitivity, extremely small size, simple configuration, flexibility in tuning sensitivity and dynamic range. An intrinsic FP interferometer normally entails that the formation of the cavity is made inside of the OF, providing it with the potential for low insertion-loss and multiplexing. Independently of the type of FP configuration used [11], the cavity output signal always presents an interference pattern that is a function of the length and refractive index of the cavity. FP structures using common OF and a MOF are an ever more common structure. Since they use two different elements in the cavity, they are commonly called hybrid FP cavities. A hybrid structure that used a MOF as the guiding fiber and cascade it with a hollow-core fiber and a single mode fiber (SMF) was demonstrated for high-temperature sensing [12]. A hybrid FP based on a suspended-core MOF showed a sensitivity of 9.8 pm/°C to temperature variations [13]. Two miniature hybrid FP sensors were developed for high temperature measurement: one using 72.3 μm double-core MOF as the cavity, with a sensitivity of 13.9 pm/°C [14] and, another using 48 μm of endlessly single mode MOF with a sensitivityof 11 pm/°C [15]. By inserting a hybrid FP interferometer in a laser cavity, a sensitivity of ~6 pm/°C was achieved [16]. An interrogation system based on a dual-wavelength Raman fiber laser was used to unambiguously recover the temperature of a hybrid FP based on a SMF and a suspended-core fiber, obtaining a sensitivity of ~0.84 deg/°C [17].

In this manuscript, the numerical and experimental characterization of a hybrid low-finesse Fabry-Pérot interferometer is performed for temperature sensing. The hybrid Fabry-Pérot cavity is based on a four-bridge double-Y-shape-core microstructured fiber. Studies are presented exploring the cavity’s response to temperature changes, polarization and power fluctuations over time.

## 2. Numerical Characterization

**Figure 1:**
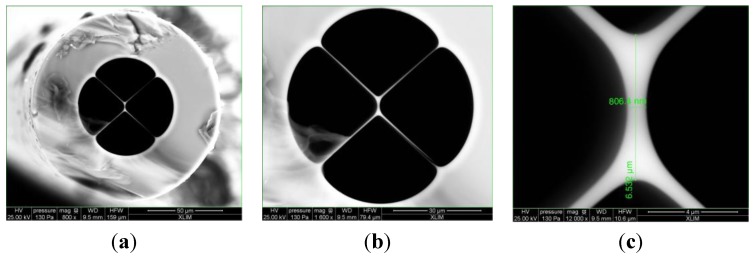
Microscope photograph of the microstructured optical fiber(MOF)’s cross section (**a**) with 800× amplification; (**b**) 1600× amplification; (**c**) core detail with 12,000× amplification.

The four-bridge double-Y-shape-core MOF used was fabricated using the stack and draw process. It is formed by four large air holes divided by four bridges, presenting a suspended core of 6.5 μm by 806 nm, exhibiting a double Y shape, as can be seen in the photos shown in Figure 1. This specific core shape can be seen as two coupled single mode guiding cores but for more accuracy, we have considered the whole structure and calculated all electromagnetic modes using the vectorial Finite Element Method (FEM).

**Figure 2:**
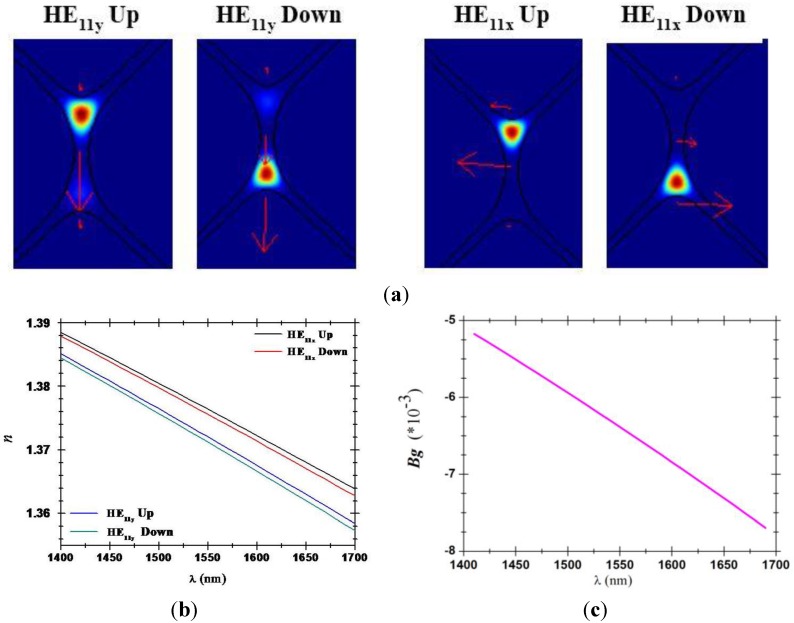
(**a**) Field distribution of HE_11*x*_ and HE_11*y*_ up and down modal components of LP_01_; (**b**) Effective refractive indices *versus* wavelength; (**c**) Group birefringence *versus* the wavelength.

The simulation results are displayed in Figure 2. The intensity distributions of two calculated electromagnetic components of LP_01_ supermodes are displayed in Figure 2a, and red arrows were added to indicate the polarization direction of each mode. The structure can propagate LP_01_ supermodes composed of electromagnetic modes we called HE_11x-up_, HE_11y-up_, HE_11x-down_ and HE_11y-down_. No higher order modes have been calculated for this structure. Figure 2b presents the refractive index variations *vs* wavelength for all calculated HE_11_ modes. It should be noticed that, for one polarization, the refractive effective indices are very close to each other, due to the symmetry of the structure. Figure 2c exhibits the computed group birefringence (*B_g_*), calculated using the “up” electromagnetic modes (results using the “down” modes are similar). The calculated *B_g_* values are similar to the ones presented in [18], which lead us to consider this fiber as a Hi-Bi MOF. For all these reasons, specific attention has been paid to polarization and launching conditions during the experimental work. The impact of launching conditions will be discussed in the next section.

## 3. Experimental Characterization

Using the four-bridge double-Y-shape-core MOF, a low-finesse Fabry-Pérot interferometer was fabricated. An experimental study was performed for this cavity in order to observe its response with temperature, polarization and power fluctuations over time.

### 3.1. Fabry-Pérot Cavity Fabrication

**Figure 3:**
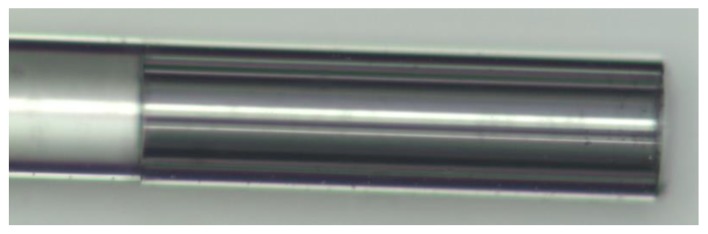
Microscope photograph of the Fabry-Pérot cavity fabricated.

The Fabry-Pérot cavity was made by splicing a single mode fiber to one side of a four-bridge double-Y-shape-core MOF (in Figure 1). The MOF was cleaved at the other end, leaving the cavity with a length of ~500 μm. Figure 3 shows the photograph of the resulting Fabry-Pérot cavity. The splice was made using a conventional arc-electric fusion splicer machine, in manual mode. Since this MOF has high air filling fraction, the typical arc power and duration used for SMF-SMF fusion induces the MOF to collapse. As so, a study was made on the arc power and duration settings in order to develop a new adapted program. This program allowed splicing the SMF and MOF without collapsing the MOF, through a series of 20 splices with very low arc power and duration. To insure that the SMF and MOF’ cores were aligned before splicing, the manual approximation of both fibers was made while illuminating the SMF and retrieving the signal at the MOF’s other end. Through the output at the MOF it was possible to observe the amount of light transmitted from one core to the other.

A low-finesse Fabry-Pérot interferometer consists of two mirrors separated by a cavity of length d. This cavity of length d can be made by a MOF, since its core refractive index is different from the SMF’s core refractive index. When splicing a piece of MOF to a SMF two mirrors are formed at the extremities of the MOF: (1) in the interface SMF-MOF due to the discontinuity in refractive index between both fibers and (2) at the interface MOF-air, since this high discontinuity provides Fresnel reflection (3.3%). The low-finesse Fabry-Pérot interferometer is created when a light beam enters the cavity (MOF) and is reflected multiple times between the interfaces (1) and (2). Each beam has a fixed phase difference with respect to the preceding one, and this phase difference corresponds to the extra path length travelled in the cavity. The interferometric signal of this cavity has a period corresponding to ∆λ = λ^2^/(2nd), where λ is the wavelength of operation and n is the cavity refractive index.

### 3.2. Experimental Setup

**Figure 4:**
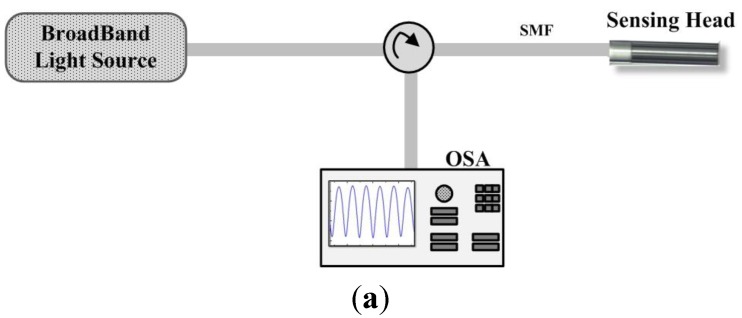

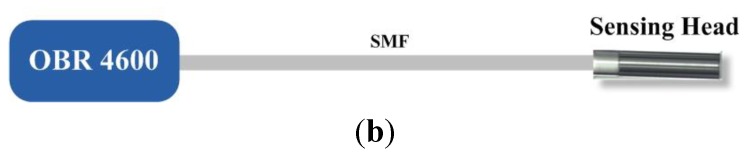
Illustration of the experimental setups used to characterize the Fabry-Pérot interferometer. (**a**) Using a broadband light source; (**b**) Using an optical backscatter reflectometer (OBR).

The Fabry-Pérot cavity sensing head was characterized using two different setups, presented in Figure 4. In one setup, in Figure 4a, illumination was provided through a broadband light source, working in the L-band, and a circulator, being the output signal collected by an optical spectrum analyzer (OSA) Advantest Q8384. In the other setup, in Figure 4b, light from an optical backscatter reflectometer (OBR) 4600 Luna Technologies, was injected into the FP cavity, and collected back in to the OBR. The OBR uses swept-wavelength coherent interferometry to measure minute reflections in an optical system as a function of the wavelength.

For the polarization measurements, an optical polarization controller was added between the illumination source and the sensor head, in both configurations.

## 4. Results and Discussion

**Figure 5:**
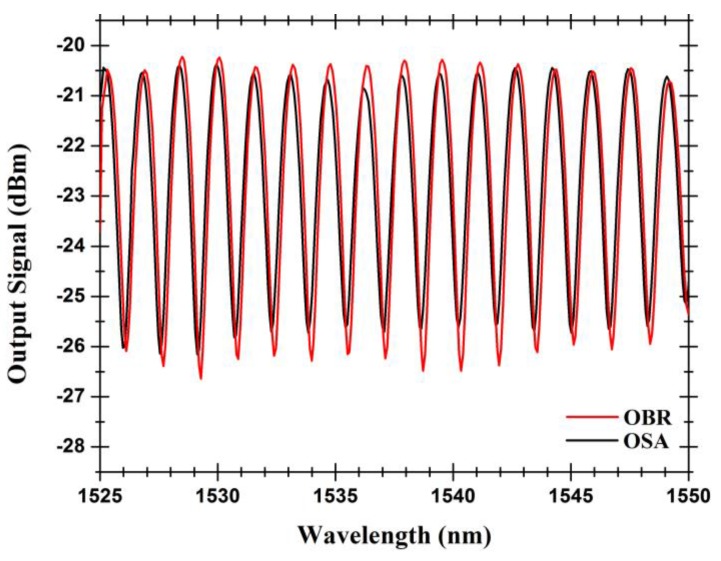
Output signals of the hybrid Fabry-Pérot cavity measured by the two different optical instruments (optical spectrum analyzer (OSA) and OBR).

The obtained output signal of the Fabry-Pérot cavity, when measured by two different setups, can be seen in Figure 5. The low-finesse Fabry-Pérot interferometer presents an interferometric fringe pattern with a wavelength spacing of 1.6 nm. It must be stated that such a pattern is observed when the coupling conditions provide a balanced distribution of power between the “up” and “down” components of LP_01_ mode (*i.e.*, when modes are excited with the same power because the SMF core is aligned with the MOF’s core). When the fiber’s cores are misaligned, secondary interferences pattern can be observed due to beating between modes.

The value of 1.6 nm for the periodicity of the channeled spectrum shown in Figure 5 corresponds to the interference between the light beam reflected in the interface between the input single mode fiber and the MOF fiber and the beam originated by the Fresnel reflection at the far end of the MOF fiber. Other factors that affect the periodicity of this spectrum are namely the difference of the propagation constants of the up and down HE_11_ modes and, in each of them, its x and y polarizations (Figure 2c), that have a much smaller influence as a consequence of the associated residual optical path differences when compared with the value of 2 × 500 × 1.5 = 1500 μm, which is the optical path difference between the two waves above identified.

### 4.1. Response with Temperature

**Figure 6:**
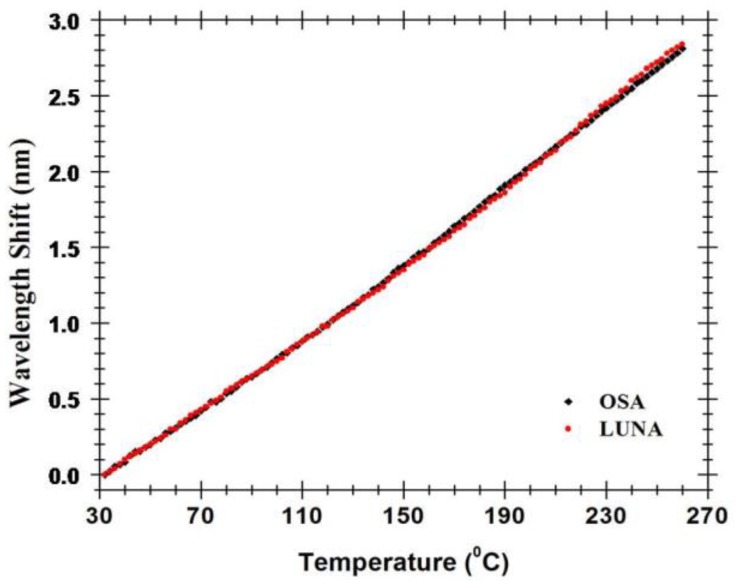
Wavelength shift of the hybrid low finesse Fabry-Pérot interferometer with the temperature.

When temperature variations are enforced into the FP cavity, its interferometric signal suffers a wavelength shift. In order to characterize this wavelength shift, the FP cavity was placed in an oven working in a temperature range of 30 to 270 °C. The observed wavelength shift with temperature is presented in Figure 6. Within a peak wavelength range (~1.6 nm), the sensor presents the ability to accurately and unambiguously measure 90 °C.

The Fabry-Pérot cavity presents a linear response with temperature with a sensitivity of 12.5 pm/°C, for both setups. The sensitivity of this FP cavity is higher than the one of the mature fiber Bragg grating technology (typical sensitivity of 8.4–10.6 pm/°C) [19], and then other MOF based hybrid structures [7]. This sensitivity might be due to the symmetric structure of the four-bridge double-Y-shape-core MOF and the coupling conditions of the Fabry-Pérot cavity. This four-bridge double-Y-shape-core microstructured fiber’s sensitivity to temperature, allied with its ability to monitor volatile compounds as presented in [20], opens up the possibility for simultaneous temperature and gas sensing through the low-pass frequency filtering technique [21]. Moreover, real-time monitoring of several of this sensing heads multiplexed can be accomplished by using FFT analysis and a precise interrogation technique [22].

### 4.2. Response with Polarization

**Figure 7:**
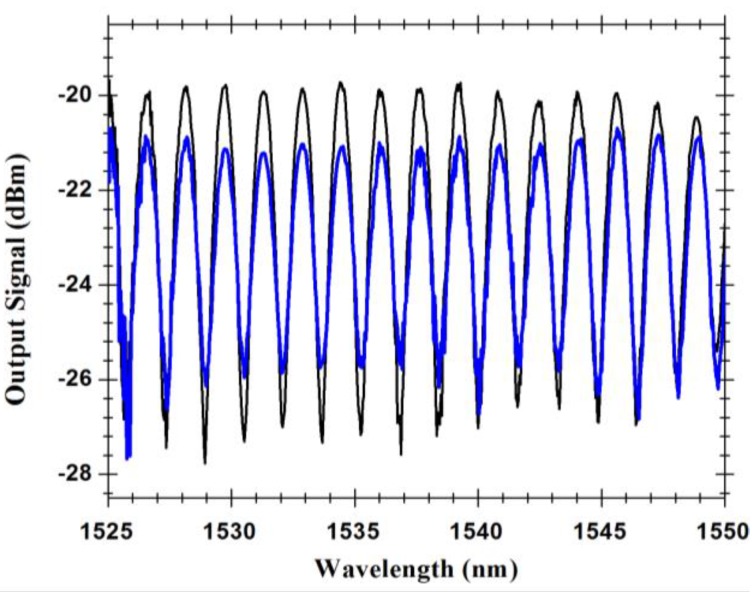
Output signal for different polarization states. **Black line**: Maximum polarization state; **Blue line**: Minimum polarization state.

Another interesting characteristic of this Fabry-Pérot sensing head is its sensitivity to polarization changes. When illuminating the Fabry-Pérot cavity with polarized light and changing the polarization controller state, different interference dynamic ranges were found, as can be seen in Figure 7. It can be observed that the polarization of the illumination light seems not to affect the location of the maxima and minima of the interferometric spectrum. As mentioned before, the reason stands on the fact that the periodicity of the observed spectrum is, to a rather large extent, determined by the optical path imbalance associated with the extension of MOF fiber (500 μm), whatever of the *x* or *y* polarization states of the illumination light. In other words, the birefringence of the MOF fiber has a second order impact in the proposed configuration. Anyway, by careful examination of Figure 7 it can be observed that the fringe maxima of the interferometer corresponding to the two polarizations coincide in the left side wavelength region (around 1525 nm), but some dephasing can be noticeable in the other end (1550 nm), a consequence of the different effective refractive indices (birefringence) connected with the two polarizations.

**Figure 8:**
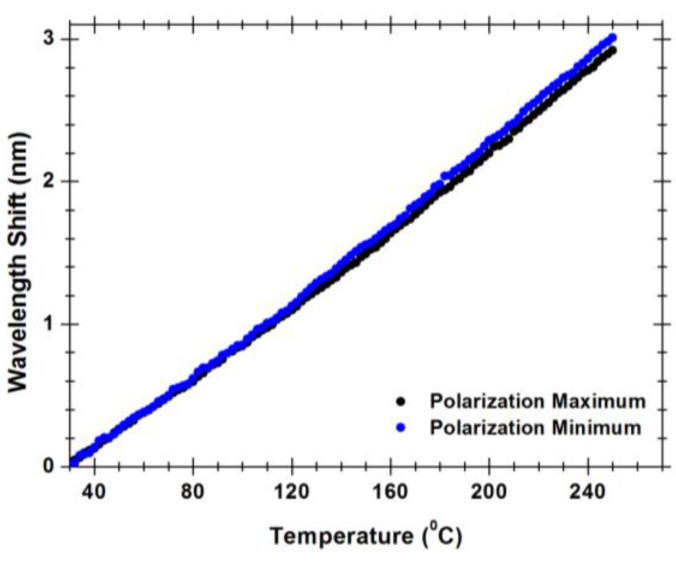
Wavelength shift of the hybrid low finesse Fabry-Pérot interferometer with the temperature for polarization maximum and minimum.

Anyhow, Figure 7 also shows that the polarization of the illumination light has a noticeable effect on the visibility of the interferometer, with the peak-to-peak amplitude of one fringe pattern being smaller compared with the other related with the orthogonal polarization. Observing Figure 1 and Figure 2a, it is clear the situations of *x* or *y* polarization of the propagating light are not equivalent concerning losses, considering in one case the light wave propagates essentially in glass, while in the other situation there is propagation in glass and air via evanescent field, this asymmetry is also the source of the fiber birefringence. This feature has influence on the measurement dynamic range. The measurement of temperature is made by measuring each point (wavelength, output power) of the interferometric spectrum for each temperature. A maximum visibility of 8 dB was measured for the best polarization state using the OSA. However, for the worst polarization state, the visibility can be as lower as 3 dB. As can be seen in Figure 8, the measured wavelength shift for best and worst polarization states only changes 0.4 pm/°C. This allows sensor customization, since through the polarization control we can optimize the output sensor properties.

### 4.3. Response over Time

**Figure 9:**
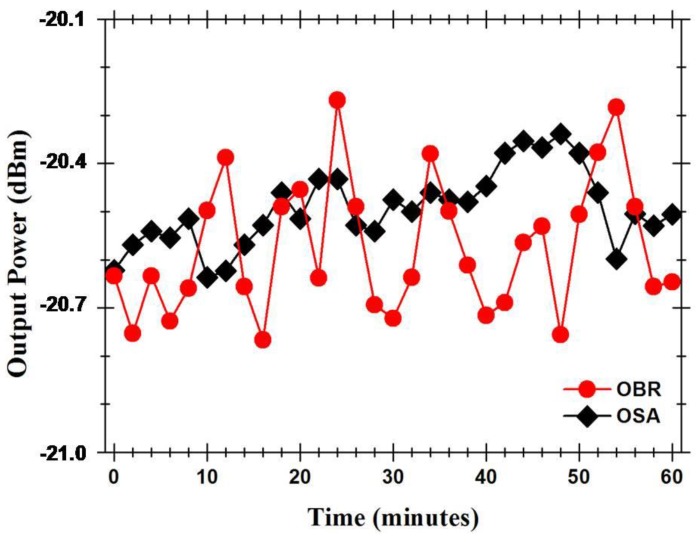
Output signal’s power fluctuations over time.

**Figure 10:**
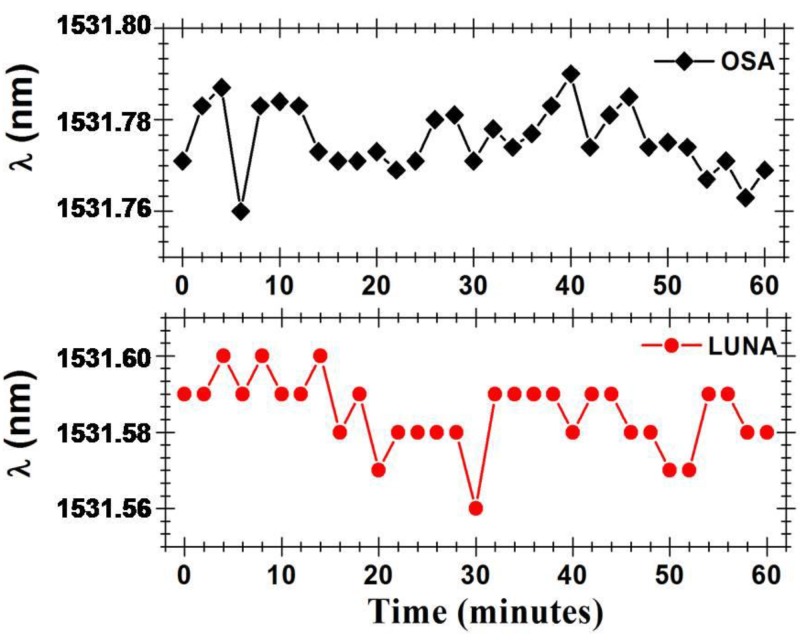
Output signal’s wavelength fluctuations over time.

With the intention of determining the Fabry-Pérot cavity sensing head output signal’s stability, the power and wavelength fluctuations of this cavity were measured during one hour, in time intervals of 2 min. Figure 9 presents the optical power fluctuations and Figure 10 presents the wavelength fluctuations of the Fabry-Pérot sensing head using each of the setups presented in Figure 4. In a time window of one hour, the Fabry-Pérot cavity sensing head presented maximum power variations of ~0.297 dB and ~0.498 dB when measured with the setups of Figure 4a,b, respectively. Regarding the wavelength response over time, it was observed that the maximum wavelength variations were of ~30.4 pm and ~39.76 pm when measured with the setups of Figure 4a,b, respectively.

## 5. Conclusions

A hybrid low-finesse Fabry-Pérot interferometer based in a four-bridge double-Y-shape-core MOF was characterized for temperature sensing. The Fabry-Pérot cavity sensing head was made by splicing a SMF to a piece of MOF (with its end cleaved). The characterization of this cavity was made using two different setups, one based in an OSA and another based in an OBR. The cavity interference pattern presented a wavelength spacing of 1.6 nm, which shifted linearly when temperature changes where induced on it. The Fabry-Pérot hybrid cavity showed accurate measurement of temperature in a 180 °C range, with a sensitivity of 12.5 pm/°C; as well as maximum power fluctuations of 0.297–0.498 dB and maximum wavelength fluctuations of 30.4–39.76 pm, in one our readings. Due to the cavity birefringent behavior, it presents sensitivity to polarization changes. Besides its temperature sensing application, this low-finesse Fabry-Pérot interferometer is also suitable for other applications such as gas sensing, fiber lasing (by using the Fabry-Pérot sensor as a mirror in the laser cavity), and wavelength division multiplexed systems by taking advantage of the interferometric signal.
